# Conference Reports: FIFTIETH ANNUAL CONFERENCE ON PHYSICAL ELECTRONICS Gaithersburg, MD June 11–13, 1990

**DOI:** 10.6028/jres.095.055

**Published:** 1990

**Authors:** Richard Cavanagh

**Affiliations:** Surface Science Division, Center for Atomic, Molecular, and Optical Physics, National Institute of Standards and Technology, Gaithersburg, MD 20899

The National Institute of Standards and Technology (NIST) and The University of Maryland jointly hosted the Fiftieth Annual Conference on Physical Electronics, held at NIST in Gaithersburg, MD, June 11–13, 1990. This annual conference provides a forum where important new research results concerned with the physics and chemistry of solid surfaces presented in a 3-day conference with ample time for discussion.

## 1. The Conference

The 1990 meeting in Gaithersburg marked both the fiftieth anniversary of the conference and the first return to the NIST site since the 1971 conference. Approximately 125 participants registered for the meeting, representing a broad spectrum of the scientific community. Participants included representatives from industry (AT&T, IBM, AMP, Perkin Elmer Corp., and Exxon), national laboratories (NRL, Sandia, Brookhaven), numerous American academic laboratories, and several international institutions (Indian Institute of Technology, University of Cambridge, University of Toronto, University of Tokyo, Forschungszentrum-Jülich, and Kyoto University).

### 1.1 Nottingham Competition

The competition to select the outstanding student paper saw an unusual number of highly qualified contestants. Their papers reflected the breadth of interests of the overall conference, covering topics from molecular beam probes of chemical processes at surfaces to the use of scanning tunneling microscopy (STM) to characterize the interaction of steps and defects. This year’s Nottingham Prize was awarded to Yuan-Wo Mo, from The University of Wisconsin, Madison. His paper, “Scanning Tunneling Microscopy Studies of Surface Kinetic Processes at the Atomic Level,” focused on the direct use of STM imaging of surface atoms to determine self-diffusion parameters.

### 1.2 Atomic-Scale Mechanisms Underlying Surface Behavior

A variety of talks at this year’s meeting continued the strong tradition of demonstrating the atomic-scale mechanisms underlying surface behavior. The increasing power of field ion microscopy to determine fundamental parameters was demonstrated in a number of talks (T. Tsong— Penn State, S. Wang—Univ. of Ill.). These experimental studies were nicely complemented by theoretical studies which utilized either first-principles techniques (P. Feibelman—Sandia) or embedded atom calculations (M. Daw—Sandia) to extract diffusion barriers, binding energies, and cluster geometries. The difficult problem of concerted surface diffusion was addressed in several experimental talks showing the use of Low Energy Electron Diffraction (LEED) studies of ordering (M. Tringides—Iowa State), laser-induced desorption studies of filling (M. Arena—Stanford), and Low Energy Electron Microscopy (LEEM) studies of step motion (R. Phaneuf—Univ. of Maryland).

The physical properties and growth properties of thin films were the focus of a group of talks, ranging from the growth of Au on Ag(110) (P. Fenter—Rutgers) to the electronic properties of metal/semiconductor interfaces such as Cs-GaAs(110) (T. Wong—Univ. of Penn.) and Cs-InSb(110) (L. Whitman—NIST).

### 1.3 Electronic Structure of Surfaces and Interfaces

A broad range of probes is now being applied to characterize the electronic structure of surfaces and interfaces. Of particular note was the emergence of laser-based three-wave mixing techniques which are now beginning to provide complementary data to that available from synchrotron-based photoemission (J. Hamilton—Sandia, L. Urbach— Univ. of Penn., S. Janz—Univ. of Toronto). Several related papers reported on the role played by these excited surface electronic states in radiation-induced surface reactions, ranging from hot electron attachment to Mo(CO)_6_ (Z. Ying—Cornell) to the influence of coadsorbates on excited state lifetimes (T. Orlando—Sandia).

With an eye toward future device developments, the layer-dependent properties and electronic excitations in cleaved high temperature superconductors were discussed (J. Demuth—IBM) as were the kinetic and dynamic factors associated with ballistic electron emission across interfaces (M. Stiles— NIST).

### 1.4 Advances in Electron Spectroscopies

Recent refinements in both instrumentation and interpretation of x-ray photoelectron spectroscopy (XPS) lineshapes have led to the use of core-level shifts to follow both reconstructions (G. Wertheim—AT&T) and the degree of charge transfer at interfaces (Y. Ma—AT&T). Parallel advances in electron spectroscopies have permitted the clear identification of adsorbate symmetry at surfaces (F. Sette—AT&T), measurement of substrate dielectric response (E. Jensen—Univ. of Cambridge), and direct probing of adsorption/desorption kinetics at constant coverage (B. Hinch—AT&T, L. Peterson—Univ. of Oregon). The elegant use of synchrotron-based angle-resolved photoemission to map out surface Fermi contours of O/W(011) and O/Mo(011) and to establish the relationship of these states to the surface reconstruction (S. Kevan—Univ. of Oregon) was discussed. Results from an exciting new tool for the study of electronic state densities (Auger Coincidence Spectroscopy) were reported for the TaC(111) surface (R. Bartynski—Rutgers).

### 1.5 Equilibrium Statistical Mechanics

Studies of equilibrium statistical mechanics at surfaces were represented by direct STM imaging of step wandering (X. Wang—Univ. Maryland) and related calculations (T. Einstein—Univ. of Maryland), by a LEED search for the elusive surface roughening and/or melting transition (Y. Cao—Univ. of Missouri), and by an x-ray study of soliton pinning in an epitaxial overlayer (K. Liang—Exxon).

### 1.6 Molecular Processes at Surfaces

Remarkable progress was noted in the frontier of molecular processes at surfaces. Several theoretical talks addressed the nature of molecular energy transfer at surfaces using techniques such as wavepacket dynamics to account for the redistribution of energy following molecular beam scattering (N. Sathyamurthy—Indian Inst, of Tech.), electron stimulated desorption (D. Jennison—Sandia), and laser induced processing (H. Guo—Northwestern Univ.). These theoretical reports were complemented by several timely experimental papers which included femtosecond probes of adsorbate energy transfer processes (J. Beckerle—NIST), optical, electron and atom scattering probes of the vibrational dynamics of ideally H-terminated Si(111) (Y. Chabal—AT&T), and state-resolved measurements of non-thermal desorption phenomena (L. Richter—NIST, T. Orlando—Sandia). In addition, there were several papers which reported on optically driven surface reactions where excited carriers in the substrate were identified as being responsible for the observed processes (L. Richter—NIST, Y. Li—Univ. of Calif. Irvine, Z. Ying—Cornell).

Molecular beam scattering techniques were used in several experiments to clarify the adsorption/absorption process. Collision induced absorption in the H/Ni(111) system demonstrated the role of high-kinetic-energy collisions in driving adsorbates into the subsurface (A. Johnson—MIT). This result is particularly significant in terms of accounting for reaction pathways which may only contribute at elevated pressure. Molecular beam scattering was also used to probe the surface/subsurface kinetics of H/D exchange on Pd surfaces (V. Shamamian— Sandia) and trapping-mediated chemisorption of ethane (C. Mullins—Cal. Tech.)

### 1.7 Magnetism

Characterizations of the magnetic properties of bulk metals and thin epitaxial films were presented in several papers. Bulk materials [Mo(110) and Cu(100)] were probed using spin-exchange electron scattering (G. Mulhollan—Rice Univ.). Thin films of Co/Cu(111) and Fe/W(100) were examined using the surface magneto-optic Kerr effect (M. Kief—Penn State) and spin-polarized angle-resolved photoemission (R. Fink—Univ. of Texas), respectively.

## 2. Summary

A healthy balance between theory and experiment was reflected in the papers at this year’s meeting. The diversity of topics and the quality of the papers reflect the vitality of surface science.

